# HSV-1 tegument protein and the development of its genome editing technology

**DOI:** 10.1186/s12985-016-0563-x

**Published:** 2016-06-24

**Authors:** Xingli Xu, Yanchun Che, Qihan Li

**Affiliations:** Institute of Medical Biology, Chinese Academy of Medical Sciences, Peking Union Medical College, Kunming, Yunnan China

**Keywords:** HSV-1, Tegument protein, Homologous recombination, BAC, CRISPR/Cas9 system

## Abstract

Herpes simplex virus 1 (HSV-1) is composed of complex structures primarily characterized by four elements: the nucleus, capsid, tegument and envelope. The tegument is an important viral component mainly distributed in the spaces between the capsid and the envelope. The development of viral genome editing technologies, such as the identification of temperature-sensitive mutations, homologous recombination, bacterial artificial chromosome, and the CRISPR/Cas9 system, has been shown to largely contribute to the rapid promotion of studies on the HSV-1 tegument protein. Many researches have demonstrated that tegument proteins play crucial roles in viral gene regulatory transcription, viral replication and virulence, viral assembly and even the interaction of the virus with the host immune system. This article briefly reviews the recent research on the functions of tegument proteins and specifically elucidates the function of tegument proteins in viral infection, and then emphasizes the significance of using genome editing technology in studies of providing new techniques and insights into further studies of HSV-1 infection in the future.

## Background

As a viral disease with an enormous impact on human health, herpes simplex virus 1 (HSV-1) infection typically generates uncomfortable, watery blisters on the skin or on mucous membranes of the mouth and lips [1, 2] and potentially leads to encephalitis with remarkable sequelae or vesicle eruption on genital organ, with an increasing incidence in recent years [3]. Importantly, the eruption of these blisters and vesicles are frequently attributed to the long-term latent infection of HSV-1 in the nervous system [4]. Instead, the clinical symptoms of acute infection, as well as the long-term pathologic processes induced by recurrent latent infection, have been shown to closely correlate with the complex viral genome structures and the molecular mechanism of viral gene transcriptional regulation and replication [5, 6].

The basic structure of HSV-1 consists of four elements: the core and capsid are composed of a double-stranded DNA genome and viral DNA binding proteins surrounded by an icosahedral capsid; the tegument is a layer between the capsid and the envelope; the envelope is the outer layer of the virion and is composed of an altered host membrane and a dozen unique viral glycoproteins. Many previous studies on viral structures have demonstrated that the tegument proteins are a group of viral structural components playing an important role in viral gene replication and virion assembly [7]. Many researchers have undertaken substantial studies on tegument proteins for a long time. In the 1970s, Abodeely et al. isolated a complete and purified HSV-1 particle from hamsters with viremia and subsequently identified 20 structural proteins by polyacrylamide gel electrophoresis (PAGE) [8]. Spear and Heine et al. identified 24 structural proteins by optimizing the viral purification assay [9, 10], of which vhs and VP16 were first defined for the first time due to their essential functions in viral infection and gene transcriptional regulation [11, 12]. Subsequently, several tegument proteins associated with viral DNA transport into and out of the nucleus for viral assembly have been identified: VP22 with a crucial role in viral gene transcriptional regulation and virion capsid formation and envelopment [13]; UL36 involving in the targeting of incoming capsids to the nucleus and the release of the viral genome into the nucleus [14]; UL31, Us3, ICP34.5, UL36, UL37 and UL51 required for regulating viral nuclear egress; other proteins, including UL36, UL37, UL11, UL20 and UL46-49, associating with the primary envelopment of HSV-1.

In the 1970s, the establishment and development of protein mass spectrometry rapidly promoted and facilitated further studies on tegument proteins, which thereby largely contributed to the identification of several proteins with small content or low molecular weight. Loret et al. identified four tegument proteins—UL7, UL23, UL50 and UL55—by mass spectrometry in 2008 and concomitantly confirmed the feature of tegument proteins for ICP0 and ICP4, regardless of the long term argument for their biological features [15]. Thereafter, the function of UL7 was determined to be closely associated with the virulence and virion assembly and egress from the envelope [16, 17]. UL50 was seen to play an essential role in neurovirulence induction and in nucleotide metabolism [18]. Although the network of viral protein interactions has been gradually established for clarifying the function of multiple tegument proteins via the use of many assays, such as biochemical analyses, yeast two hybrids and co-immunoprecipitation [19], research on the immune response in humans to viral infection has just been initiated. An increasing number of studies have demonstrated the crucial roles of multiple tegument proteins, including ICP0 and Us3 in inducing interferon production and subsequently activating the immune response in hosts. Collectively, these findings appear to suggest the potential role of the tegument proteins in the development of new vaccines and drugs for protection from viruses and in the treatment of viral diseases. In this article, the function of the HSV-1 tegument proteins is briefly described for a better understanding of their potential application in clinical studies.

### Early studies on tegument proteins

vhs is first identified as one of the tegument proteins for playing an important role in the early stage of viral infection in cells. Nishioka et al. discovered that HSV infection in Friend erythroleukemia cells (FL cells) could lead to the inhibition of globulin synthesis and the degradation of globulins encoding RNA [20, 21]. Schek et al. observed that HSV-1 infection in Vero cells was linked to the degradation of actin, tubulin and histone mRNAs [11]. With the development of viral DNA mutation technology, genomics and proteomics, Oroskar et al. identified a temperature-sensitive mutant strain with greatly improved cellular mRNA stability during infection [22]. This finding was subsequently confirmed to be related to the vhs tegument protein [23]. The vhs tegument protein was found to function in the cellular mRNA degradation process at an early stage of viral infection. In the next ten years, an increasing number of functions of the vhs protein have been described as follows: the vhs protein is a tegument protein of 58 kDa encoded by the UL41 gene and enable of entering cells to induce mRNA degradation; vhs is subsequently responsible for guiding cells from a state of host protein synthesis to viral protein synthesis; the vhs protein has been shown to consecutively promote the transcription of viral mRNA and to facilitate the maintenance of the expression of viral genes after the initiation of viral gene transcription [24]. Everly and Read discovered the biological features of the vhs protein and its activity of an RNA enzyme for inducing RNA degradation in cells, including the degradation of viral mRNA via its presence or simply by its interaction with cellular transcriptional initiator 4H [25]. In infected cells, vhs exhibits a specific selection for terminal mRNA molecules and first triggers mRNA degradation in the 5′ terminal sequence of the translation initiating domain prior to the degradation of the 3′ terminal sequence [26]. In contrast, this limited characteristic of vhs is failed to exhibit in purified virions due to the absence of specific regions for RNA degradation [27]. All of these findings are most likely to imply that the potential interaction of specific cellular proteins and vhs is typically lacking in purified virions; which thereby makes the vhs failing to display the specific site selection of mRNA molecules triggered in cells [28]. In recent years, researchers also noted that mRNA degradation by vhs could potentially be regulated by its interaction with the tegument protein UL47 [29].

The function of another well-known tegument protein, VP16, in viral gene expression and regulation has been studied since the 1980s. The HSV-1 gene expression cascade in terms of immediate early, early, and late genes was demonstrated to be strictly controlled. In particular, during the early stages of a viral infection, a viral structural protein was identified to induce immediate early gene expression as a result of its presence in the cellular environment with the inactivated HSV-1 [30]. Campbell et al. co-transfected a chimeric plasmid containing individually cloned HSV-1 genomic fragments and immediate early gene-controlled thymidine kinase genes into cells for localizing the regulatory factor of this gene at the EcoRII enzyme-cutting site (the axis in viral genome is 0.635–0.721) by assaying thymidine kinase activity [12]. Further gene cloning confirmed the identity of this protein as ICP25 (now named VP16). The transcriptional regulation of VP16 on the viral IE gene was determined by a common co-motif (the sequence is TAATGARAT) with one or multiple copies in the upstream region of a specific IE initiating domain [31, 32]. Nevertheless, VP16 was not shown to individually bind to this motif nor bind to non-specific DNA sequences. Rather, VP16 played a role in transcriptional regulation via the interaction of the cellular transcriptional factor OCT-I with the octamer sequence (containing TAATGARAT motif) and the host cell factor-1 (HCF-1) [33, 34].

### The developmental studies of tegument proteins

Before the 1990s, the identification and localization of HSV-1 proteins appeared to merely depend upon mutant viral strains that were temperature-sensitive and on the marker rescue assay, which thereby led to limitations in studying its related functions. In 1992, Shizuya et al. constructed the first bacterial artificial chromosome (BAC) vector BAC108L based upon an F1 factor backbone of *Escherichia coli* with an insertion of a 300 kb DNA fragment [35]. This approach was mainly applied to identify the specific deletion, which greatly promoted the studies of HSV-1 viral gene functions. Additionally, the development and employment of mass spectrometry largely contributed to facilitating the identification of four new tegument proteins, UL7, UL23, UL50 and UL55, as well as the classification of the ICP0 and ICP4 proteins as tegument proteins [15]. These findings will be individually described in the following paragraph.

### Viral gene transcriptional regulation factor

All the Herpesviruses have been found to exhibit the cascade of immediate early genes, early genes, and late genes in viral replicative gene expression, during which multiple tegument proteins are speculated to be largely involved. When a viral genomic DNA molecule enters into the nucleus, the VP16 viral protein first initiates a gene transcriptional event to enable cis-activation of the transcription of the immediate early proteins ICP0, ICP4, ICP22, ICP27 and ICP47. Upon production of ICP0, ICP0 and the nuclear domain 10 (ND10) sub-structure co-distribute within spots in the nucleus. The viral protein ICP0 subsequently induces SUMO-1-modified PML and sp100 to damage the ND10 structure via a protease pathway within several hours and stimulate viral infection and cis-activate early gene and late gene transcription [36]. Furthermore, ICP0 is evidenced to enhance early and late gene transcription by neutralizing the inhibitory activity associated with the HDAC amino terminal by interacting with the HDAC 4, 5, 7 amino terminals [37].

Another transcriptional regulatory factor required for establishing HSV-1 infection is ICP4 responsible for modulating HSV-1 replication and transcription required for balancing the HSV-1 entry into the infectious or latent state. ICP4 is able to negatively modulate the ICP4 promoter, the latency-associated promoters and long and short junction-spanning transcripts (L/ST) promoters [38–40]. ICP4 can activate the early and late gene transcription of a vast majority of viruses by acting as an important transcriptional activating factor. ICP4 exhibits a specific affinity to DNA sequences by acting as a DNA binding protein. On the base of analyzing the known individual ICP4 binding domains, researchers summarize a group of DNA sequences with specific regularity i.e., RTCGTCNNYNYSG, of which R is purine, Y is pyrimidine, S is C or G, and N is any base [41].

Additionally, many studies have substantially evidenced the essential function of Us3 in viral gene expression and maturation. Us3 has been shown to repress histone deacetyltransferases and enhance viral gene expression via phosphorylating histone deacetylase 1 (HDAC-1) and HDAC-2 [42]. Some studies have demonstrated that UL14 could indirectly modulate immediate early gene transcription by expediting VP16 entry into the nucleus at the early stages of infection [43]. An in vitro cell culture assay revealed that the infection of the UL13 deletion mutant viral strain with cells could lead to a decrease in the expression levels of the ICP0, UL26, UL26.5, UL38, vhsand Us11 [44].

### Viral genome replication-associated UTPase protein

dUTP is hydrolyzed to dUMP and pyrophosphate via dUTP enzyme catalysis. Because it is easier for DNA polymerase to introduce dUTP into the DNA strand during replication and in the generation of point mutations or strand breaks, the hydrolyzation of dUTP by the dUTP enzyme is required for the precise replication of DNA [45]. The functions of viral UL50 have gradually been got to know since the classification of UL50 as a tegument protein by Loret et al. UL50 was identified to encode the dUTP enzyme required for viral replication. dUTP is also involved in nucleotide metabolism, which plays an essential role in viral replication. Akihisa Kato et al. observed that the UL50 encoding dUTP enzyme had the capacity to interact with cellular dUTP to cooperatively expedite precise viral gene replication. Furthermore, viral dUTP enzyme activity was typically shown to depend on the phosphorylation of serine187 by the Us3 protein [18]. In the subsequent experiment, the neurovirulence of the virus was clearly eliminated, and the mutation ratio of the viral genome was significantly increased in infected mice upon serine 187 mutation to alanine. The high expression of the dUTP enzyme in cells was shown to clearly improve these biological effects, reduce genomic mutations and lead to the recovery of neurovirulence [46].

### Thymidine kinase (TK)

The HSV-1 UL23 gene encoding thymidine kinase (TK) has received great attention for a long time. However, TK was defined to be a member of the tegument protein family for the first time by protein mass spectrometry in 2008 by Loret et al. TK is responsible for synthesizing thymidine (Thd) and ATP into thymidine monophosphate (TMP) and exhibits deoxycytidine kinase (dCK) activity for phosphorylating deoxycytidine (dCyd) to dCyd monophosphate. TK also act as thymidylate kinase (TMPK) to phosphorylate TMP to thymidine diphosphate (TDP) [47, 48], as well as capable of phosphorylating acyclovir to acyclovir monophosphate, which is directly linked to the repression of viral replication by reducing DNA polymerase activity, and thereby makes TK becoming an essential target for the development of acyclovir anti-viral drugs and associated drug tolerance studies.

### Viral virulence-associated proteins

Chou et al. had previously reported that ICP34.5 was closely associated with HSV-1 neurovirulence [49]. Subsequent studies indicated that ICP34.5 could antagonize Beclin 1-mediated autophagy. ICP34.5 was shown to reduce this protective function in the host via its interaction with the mammalian autophagy protein Beclin 1 [50]. The study of viral UL14 function was performed in a mutant strain containing a UL14 deletion. UL14 is a highly conserved protein of approximately 32 kDa frequently phosphorylated and expressed at low levels in the herpes virus family. The infection of cells with the virus with a UL14 gene deletion at a low multiplicity of infection (moi) displays a slow replication speed for repressing viral release. When the mutant virus is intracranially injected in mice, the 50 % lethal dosage is shown to be reduced by over 30,000-fold, and the number of recurrent infections in mice infected with this mutant virus is remarkably reduced as well. All of these data are sufficient to implicate the essential roles of UL14 in viral replication and in the viral lytic and latent infection processes [51]. The mutant strain with the UL7 gene deletion exhibits equally reduced replication and plaque formation in Vero cells, which appears to suggest the potential function of UL7 in viral virulence [16].

### Viral transport and maturation-associated proteins

About half of the HSV-1 encoding tegument proteins play essential roles in viral transport and maturation. When HSV-1 attaches to cells, the majority of tegument and envelope proteins are triggered to release from the viral nucleocapsid. However, only a small number of tegument proteins still bind to the nucleocapsid and subsequently migrate into the nucleus. Us3, UL36 and UL37 are not identified to release from the nucleocapsid. Us3 is another protein kinase of HSV-1 that encodes a serine/threonine kinase. Many studies have shown substantial evidence of the crucial function of Us3 in viral gene expression and maturation. Us3 was shown to not only expedite nucleocapsid transport from the nucleus to the cytoplasm [52] but also to ensure the fusion of the viral envelope with the peripheral nuclear membrane during viral egress from the nucleus by phosphorylating the gB protein [53]. The UL36 protein is a highly conserved tegument protein with low expression levels in the herpes virus family. The study of UL36 started from the construction and phenotype analysis of the tB7 temperature-sensitive mutant viral strain, but the study remained stagnant until the construction of the mutant viral strain with the UL36 deletion. The phenotype identification assay revealed that UL36 deletion could directly contribute to viral nucleocapsid aggregation in the cytoplasm and subsequently repressed capsid formation and envelopment [54]. The study of UL36 localization showed a characteristic peripheral distribution of UL36 at an early stage of viral infection, with de novo-synthesized UL36 being distributed in the peripheral nucleus, cytoplasm and rarely in the nucleus. The findings of nucleocapsid aggregation in the mutant strain with a UL36 deletion, as well as the characteristic distribution of UL36 proteins are most likely to imply that UL36 might be a critical factor in expediting nucleocapsid transport to the cytoplasm for tegument formation [55]. Importantly, the expedition of UL36 transport to the nucleocapsid is speculated to mainly depend upon the highly conserved nuclear localization sequences in the neighboring N-terminal structural domain [56]. Additionally, UL36was shown to promote the secondary envelopment in virion assembly through its direct interaction with the UL37 protein in the N-terminal structural domain [57].

However, most of the tegument proteins have been shown to release from the nucleocapsid upon attaching to cells and subsequently to perform their functions in viral envelopment. UL11, UL6, UL21, UL47, VP16 and VP22 are closely associated with viral envelopment. The mutant viral strain with the VP22 deletion exhibits a remarkable repression on egress from the nucleus and subsequent spread between cells, which allows for an implications of several VP22 functions in virion assembly and cellular egress with mechanisms remained largely unknown [58]. VP16 is a classical transcriptional regulatory factor with a crucial role in virion assembly. A substantial nucleocapsids are aggregated in the cells infected with the viral strain with the VP16 gene deletion, and an increase in the virion with incomplete envelopes is observed [59]. The virion with the incomplete envelope produced in cells infected by the viral strain with the UL11 gene deletion is increased by 2-3-fold compared to cells infected with a wildtype strain [60]. Currently, there are few reports on the studies of UL16, UL21 and UL47, and UL16 is speculated to involve in envelopment as a result of its direct interaction with gE proteins composed of essential envelope elements [61]. UL21 is presumed to associated with viral maturation because of the equally decreased titers and small plaques observed in the mutant strain with the UL21 gene deletion [62]. Zhuoming Liu et al. demonstrated that the number of primary enveloped virions was reduced in the perinuclear space, whereas the number of nucleocapsids was clearly increased upon infection by the mutant strain with the UL47 gene deletion. Thus, these findings are sufficient to suggest the correlation to the interaction between UL47 and Us3 and UL31 and UL34 in cells [63].

## New studies on tegument proteins

With the gradual understanding and revelation of potential functions of various tegument proteins in viral infection, scientists have started to emphasize the theoretical knowledge on the practical significance in clinical drug and vaccine development, of which the immune response induced in viral infections is receiving the greatest attention. Multiple members of tegument proteins are generally recognized to be critical in inducing an immune response in hosts. For example, Us3 has been shown to down-regulate the expression of major histocompatibility complex I (MHC-I) on the surface of inflammatory cells to allow viruses for escaping from the host immune response; to cooperatively interact with glycoprotein gB for rapidly repressing the activation of CD1d antigen delivery and natural killer T cells; to phosphorylate IFN regulatory factor 3 (IRF3) for repressing the production of IFN-β. The Us3 protein kinase has been shown to phosphorylate the α subunit of the INF-γ receptor for repressing the expression of the INF stimulating gene (ISG) induced by IFN-γ [64–66]. Us3 has been shown to phosphorylate serine 75 on p65 for repressing p65 nuclear trans-localization and the activation of the NF-kB pathway and to down-regulate inflammatory cytokine IL-8 levels for subsequently repressing the immune response induced by viral infection [67]. In a study of investigating the HIV super-infection course by HSV-1, the functions of the finger-ring structure of ICP0 and the ubiquitin ligase E3 were shown to be involved in a number of signaling pathways in cells. ICP0 has the capacity to block the anti-viral response induced by interferons during the early stages of viral infection through the specific inhibition of the cellular response mediated by interferon-regulated factor 3 (IRF3) and IRF7 [68].

Additionally, in the research and development on preventive or treatment vaccines, more and more tegument proteins have been demonstrated to be potential targets for constructing live attenuated vaccines. vhs, ICP0, ICP4, ICP34.5, UL14 and UL7 are associated with host mRNA degradation, viral gene transcriptional regulation and viral virulence rather than being required for viral growth, which are equally speculated to be good targets. The mutant strains with deletions in these genes have successfully been constructed and exhibit remarkably reduced viral pathogenicity by using the classical BAC approach. Nevertheless, the virulence of these mutants has not been sufficiently eliminated to receive attention. Notably, the Zhang Lab first reported on a CRISPR-Cas9 system that was originally observed in bacteria and archaea as an adaptive microbial immune system mediated by a long-term selection pressure of bacteriophages [69]. This technology brings new hope for precise editing of DNA viruses and the development of HSV-1 vaccines. The CRISPR-Cas9 system consists of segments of prokaryotic DNA identified by complementary base pairing of short CRISPR RNAs functioning as guiding strands to direct the Cas9 nuclease to the complementary invading DNA. Subsequently, homologous recombination or non-homologous end joining is induced to edit the target DNA. The discovery of the CRISPR-Cas9 system has provided a powerful tool for scientists to specifically target the efficient modification of the invading DNA genome. For the first time, Yan Wei and TadahiroSuenaga et al. used the CRISPR- Cas9 system to conduct the precise editing of a DNA virus with a complicated genome structure at high efficiency without off-target effects [70, 71]. Wang J et al. attempted to knock out the EBV (Epstein-Barr virus) latency-associated gene by using the CRISPR-Cas9 system for the potential treatment of latent EBV infection. Rajia cells isolated from patients with latent EBV infections and Burkitt’s lymphoma were treated with CRISPR-Cas9-associated plasmids, and the cell replication and EBV load in cells, respectively, were shown to be remarkably reduced [72]. These findings are most likely to suggest the potential application of the CRISPR-Cas9 system in vaccine development and in the treatment of viral diseases.

## Conclusion

With regard to tegument proteins, studies on the UL11, UL16, UL21, UL7 and UL55 proteins have primarily been initiated. In light of the undertakings on herpes virus family members, such as VZV, EBV and HCMV, the modification of the viral genome and the proteins associated with using various technologies have greatly promoted virus-associated research. In the 1980s, scientists could only modify viruses by using temperature-sensitive mutations and homologous recombination assays, with the subsequent identification of target functional proteins via the marker rescue assay. Thus, the studies of HSV-1-associated proteins have been restricted to viral proliferation or replication-associated molecules, in which the essential functions of the tegument proteins ICP0, ICP4, vhs and VP16 in viral gene transcriptional regulation are defined.

Despite the difficulties in construction, the time-consuming process, and the low mutation rate, the BAC system has been applied to almost all of the viral proteins. This system has played the most important role in HSV-1 studies. The vast majority of mutant viral strains with tegument deletions have successfully been constructed for the past 20 years since the establishment of BAC system. Together with the development of yeast two hybrids, pull-down assays, co-immunoprecipitation and chromatin co-immunoprecipitation technologies, the functions and mechanisms of viral gene transcriptional regulatory factors, viral virulence associated proteins, viral dUTP enzymes, viral transport and maturation-associated proteins have primarily been described and defined. However, due to the restriction to low efficiencies and high difficulties in the BAC system, the mutant viral strains with two or multiple gene deletions are still difficult to be constructed, which simultaneously leads to a restriction in the construction of viral protein interaction networks and a comprehensive understanding of HSV-1 proteomics.

Surprisingly, the development of the CRISPR-Cas9 system provides a new technique for studying HSV-1 proteins, enabling the generation of precise mutations in the HSV-1 gene within a short period of time. Furthermore, the experiment is simple and will not leave any trail of the viral genome when the genetic mutation is performed. Currently, a mutant viral strain with a point mutation and gene deletion have been successfully constructed by using the CRISPR-Cas9 system, and the function and correlated mechanisms of this protein have been subsequently explored by using pull-down and chromatin co-immunoprecipitation assays. In future studies, scientists may be able to construct mutant viral banks with multiple genetic mutations of interest, which could not only extensively be used to further analyze protein functional mechanisms and protein interaction networks but also to provide new exploratory and thinking approaches for preventing and treating against HSV-1 infections that pose a great threat to human health.
